# Performance control study of interleaved meltblown non-woven materials based on statistical analysis and predictive modeling

**DOI:** 10.3389/fncom.2023.1109371

**Published:** 2023-02-03

**Authors:** Hao Xu, Ji-Wei Xu, Long-Xiang Yi, Yu-Ting Yuan, Zheng-Qun Cai

**Affiliations:** ^1^School of Microelectronics and Data Science, Anhui University of Technology, Maanshan, China; ^2^Graduate School of Environment and Energy Engineering, Anhui Jianzhu University, Hefei, China; ^3^School of Foreign Studies, Anhui Jianzhu University, Hefei, China

**Keywords:** meltblown non-woven materials, correlation analysis, regression analysis, BP neural network, structural variables

## Abstract

Meltblown nonwoven materials have gained attention due to their excellent filtration performance. The research on the performance of the intercalation meltblown preparation process is complex and a current research focus in the field of chemical production. Based on data related to intercalated and unintercalated meltblown materials under given process conditions, a product performance prediction model of intercalated meltblown materials was established under different process parameters (receiving distance, hot air velocity). The structural variables (thickness, porosity, and compressive resilience), the change in product performance, and the relationship between structural variables and product performance (filtration resistance, efficiency, air permeability) after intercalation were studied. Multiple regression analysis was used to analyze the structural variables, and evaluation of the regression results were made using R2, MSE, SSR, and SST. A BP neural network prediction model for product performance was established. The BP neural network model was used to find the maximum filtration efficiency. The study provides theoretical support for regulating product performance by solving the maximum filtration efficiency using BP neural network model.

## 1. Introduction

Meltblown non-woven material is an important raw material for mask production. The meltblown method has very fine fibers, a fluffy structure after bonding by itself, high porosity and small average pore size, which has good filtration performance (Ji et al., 2003; Tanthapanichakoon et al., 2003). However, meltblown non-woven materials have very fine fibers, and their performance is often not guaranteed in the process of use due to poor compression resilience. Therefore, we created the interlayer meltblown method, in which coarse denier high curl fibers such as polyester staple fibers are inserted into the meltblown fiber stream during the traditional meltblown preparation process to produce a “Z-shaped” structure of interlayer meltblown non-woven materials. At the same grammage, the compression resilience of the product is greatly improved and the structure becomes more fluffy, it is widely used in the field of medical protection (Song et al., 2011; Hao et al., 2021).

**FIGURE 1:**
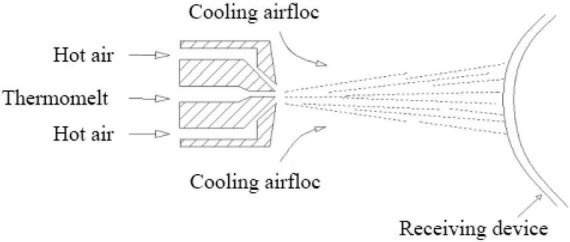
Process principle of meltblown method.

There are many parameters in the preparation process of intercalated meltblown non-woven materials, and there are interactions between the parameters, and the airflow of intercalated layers is more complicated afterward. The study of filtration resistance, filtration efficiency, and air permeability also becomes more complicated. Yesil and Bhat (2017) Investigate the effects of process variables, die temperature, air pressure, and die-to-collector distance on some characteristics of polyethylene meltblown non-wovens such as pore size, air permeability, hydrostatic head, and SEM analysis. Rathinamoorthy and Balasaraswathi (2022) aim to analyze the impact of non-woven fabric structural parameters and weathering on the microfiber release characteristics (Yesil and Bhat, 2017). Guo et al. (2011) used meltblown non-wovens as a collection material to prepare a filter material with fiber diameter grade by electrospinning technology. A brief overview of the principle of elastic non-wovens with meltblown technology and recent research progress. Čepič and Gorjanc (2022) determined the effects of spunbond and meltblown processes and various combinations of the two processes on the functional properties of medical layered non-wovens. Kwon et al. (2022) investigated the formation of superfine fibers in aquatic and air environments for 15 commercial disposable non-woven products (wet wipes) and 16 melt blown non-woven materials produced in the pilot plant, and compared them with selected textile materials and paper towel materials. Liu et al. (2020) Found that in a humid and hot environment, the meltblown material will undergo electric charge evolution, resulting in the attenuation of filtration efficiency. Dennis et al. (2020) Report that small numbers of disinfection cycles at reasonable virucidal doses of ozone do not significantly degrade the filtration efficiency of meltblown polypropylene filter material (Liu et al., 2020). Wang et al. (2022) Built on the theoretical model of film-based TENGs, here, an analytical model is introduced for conductor-to-dielectric contact-mode non-woven-based TENGs, which copes with the unique hierarchical structure of non-wovens and details the correlation between the triboelectric output (maximum transferred charge density) and non-woven structural parameters (thickness, solidity, and average fiber diameter). At present, the research on melt blown non-wovens mainly focuses on the qualitative research of process parameters and structural variables or process parameters and product performance, while the quantitative analysis of process parameters and structural variables and product performance is relatively less. In this article, the relationship models between process parameters and structural variables, structural variables and product properties are established by statistical methods such as correlation analysis, regression analysis and neural network prediction model, which provide a theoretical basis for the performance control research of intercalated melt blown non-wovens (Lan et al., 2011). As shown in Figure 1, the process principle of simplified melt spraying method.

## 2. Basic overview

### 2.1. Correlation analysis

According to the theoretical basis of the correlation analysis method it can be known that: With random variables X and Y, for which n random trials are conducted, the observations obtained are (*X*_*i*_, *Y*_*i*_) (*i* = l, 2….*n*), respectively. $\bar{X}\,\bar{Y}$ are the respective expected values of$\sqrt{D\,X}$ with$\sqrt{D\,Y}$ are the Variance of X and Y, respectively, cov (*X*, *Y*) is the co-Variance, r is the correlation coefficient, and R is the random variables X and Y for the sample (*X*_*i*_, *Y*_*i*_) (*i* = l, 2,.. *n*) of the correlation coefficient, called the sample correlation coefficient. In practice, the sample correlation coefficient R is often used as an estimate of the correlation coefficient *r*. The correlation coefficient is calculated as shown in Equation (1) (Fan et al., 2020).


(1) $$\left\{ \begin{array}{c} r=\frac{c\,o\,v\,\left(X,Y\right)}{\sqrt{D\,X}\,\sqrt{D\,Y}}\\ R=\frac{\sum_{i=1}^{n}\left(X_{i}-\bar{X}\right)\,\left(Y_{i}-\bar{Y}\right)}{\sqrt{\sum_{i=1}^{n}{\left(X_{i}-\bar{X}\right)}^{2}\,\sum_{i=1}^{n}{\left(Y_{i}-\bar{Y}\right)}^{2}}}\\ c\,o\,v\,\left(X,Y\right)=E\,\left[\left(X_{i}-\bar{X}\right)\,\left(Y_{i}-\bar{Y}\right)\right]\\ \bar{X}=\frac{1}{n}\sum_{i=1}^{n}X_{i}\\ \bar{Y}=\frac{1}{n}\sum_{i=1}^{n}Y_{i} \end{array}\right.$$

For a relationship with multiple variables, where the random variables X and Y can represent any two of the random variables.

### 2.2. Typical correlation analysis model

Typical correlation analysis is a multivariate statistical method to study the correlation between two sets of variables (multiple indicators in each set of variables), which can reveal the intrinsic connection between two sets of variables, and its core idea is to transform the correlation between multiple variables into the relationship between two representative variables.

Typical correlation analysis was proposed by Hotelling, and its basic idea is very similar to that of principal component analysis. First, the linear combination of variables in each group is identified so that the two linear combinations have the largest correlation coefficients with each other. Then the linear combination that is not correlated with the initially selected pair is selected to make it Pair, and the pair with the largest correlation coefficient is selected (Xiyuan, 2021). The selected linear Pair is called the typical variable, and their correlation coefficient is called the typical correlation coefficient. The typical correlation coefficient measures the strength of the association between these two sets of variables.

### 2.3. Back propagation neural network

Back Propagation neural network is a multilayer feedforward network trained according to the error back propagation mechanism and the forward propagation algorithm of information. The BP network can learn and store many input-output pattern mapping relationships. It can reveal the mathematical equations describing such mapping relationships after some time. Its learning rule is to use the gradient descent method to continuously adjust the weights and net values of the network by back propagation to minimize the sum of squared errors of the network, and the topology of the BP neural network model including input layer, hidden layer, and output layer (Bin et al., 2009; Liu et al., 2022).

**FIGURE 2:**
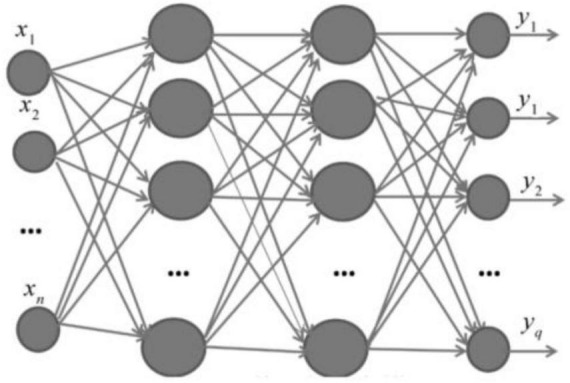
BP neural network structure.

And when a pair of learning modes is provided to the BP neural network, the corresponding input nodes are activated, the activation value propagates from the input layer through each intermediate layer to the output layer, and the input response of the network is obtained at each node of the output layer, and in the direction of reducing the deviation between the expected output and the actual output, the connection weights are corrected layer by layer from the output layer through each intermediate layer, and finally back to the input layer. With the continuous correction of this error reverse propagation, the correct rate of neural network response to the input mode is constantly improving (Horikawa et al., 1992; Liang et al., 2022). The basic structure of neural network is given as Figure 2.

## 3. Study on the law of change and related relationship after intercalation

### 3.1. The variation of structural variables and product performance after intercalation

#### 3.1.1. Descriptive statistics of structural variables and product performance

Through the descriptive statistics in Supplementary Tables 1,2, it can be preliminarily concluded that the average value of each index after intercalation increases except for filtration resistance, and the variance decreases except for air permeability.

#### 3.1.2. Trends before and after interpolation of different indicators

**FIGURE 3:**
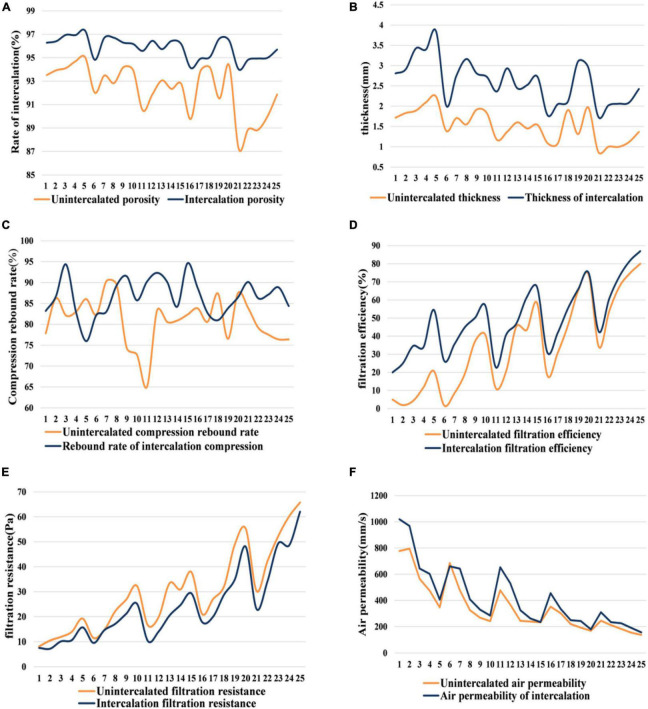
Material properties of nonwoven. **(A)** Rate of intercalation. **(B)** Thickness. **(C)** Compression rebound rate. **(D)** Filtration efficiency. **(E)** Filtration resistance. **(F)** Air permeability.

It can be obtained from Figure 3 that the Thickness, Porosity, air permeability and filtration efficiency of the non-woven materials after intercalation meltblown have been improved to a certain extent, and the filtration resistance size is reduced after intercalation melting, which is also in line with the research conclusions given by relevant literature. Namely, melt blown non-wovens are characterized by ultra-fine fibers, large specific surface area, small pore diameter and high porosity (Tanthapanichakoon et al., 2003). After intercalation, materials are added between products, so the thickness of materials will increase; In the process of intercalation, due to the characteristics of the material itself and the role of hot air, the material can be evenly distributed, effectively preventing the accumulation and blocking of the material, thus improving the porosity. Due to the improvement of the material, polyester (PET) staple fibers are inserted into the melt blown fiber stream during the melt blown preparation of polypropylene (PP), and the toughness of the material is effectively improved, so the compression resilience of the product is improved.

However, the growth of compression resilience is unstable, and there are several groups of non-woven materials inferior to unintercalated materials [It is known from fig.(f) that the compressive resilience performance of group 4–8, 18, and 20 meltblown materials after intercalation is not as good as before intercalation]. In general, intercalation can improve product performance to a certain extent, which is also in line with modern improvement technology.

### 3.2. Correlation analysis and typical correlation analysis

#### 3.2.1. Correlation of structural variables to product performance

➀ There is a significant strong positive correlation between thickness, Porosity and receiving distance, and a significant weak positive correlation with hot air velocity, that is, a proper reduction of the receiving distance can reduce the porosity and thus improve the filtration efficiency.

➁ And there is a non-significant positive correlation between Compression rebound rate and receiving distance, and a non-significant negative correlation with hot air velocity.

➂ There is a significant strong positive correlation between thickness and porosity, which is consistent with its physical properties, i.e., porosity increases with increasing thickness.

**FIGURE 4:**
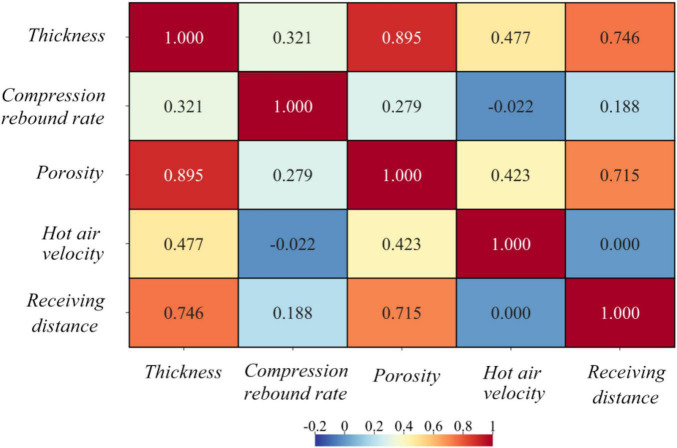
Heat map of correlation coefficient.

These can be seen from Supplementary Table 3 and Figure 4.

#### 3.2.2. The relationship between structural variables and product performance

Through the normality test (El Bouch et al., 2022), it was found that the structural variables all satisfied the normality, while the filtration efficiency and air permeability in the product performance did not satisfy the normality. Therefore, for the correlation analysis of structural variables and product performance, Pearson correlation analysis was applied to the structural variables and Spearman correlation analysis was applied to the product performance. The normality test is visible in Supplementary Table 4.

The results of correlation analysis: Within the structural variables Thickness and Porosity have a significant strong positive correlation, and Compression rebound rate has a strong negative correlation. porosity and Compression rebound rate have a significant weak negative correlation; within the product performance, there is a significant strong positive correlation between filtration resistance and filtration efficiency, and a strong negative correlation with air permeability. filtration efficiency and air permeability have a significant strong negative correlation. The results of the Spearman correlation analysis are visible in Supplementary Tables 5, 6.

#### 3.2.3. Typical correlation analysis of structural variables and product performance

The results of the typical correlation analysis solved using SPSS are as follows. It can be seen from Supplementary Table 7 that the correlation between the first two pairs of typical variables is considered to be significant through the Sig. test, and the correlation coefficient of the first pair of typical variables is 0.946. The correlation coefficient for the second pair of typical variables is 0.605.

Typical variable relationship equation.

The formula for the 1st typical variable of the set Y: Y1 = 2.537 × Thickness–0.253 × Porosity +0.043 × Compression rebound rate.
The formula for the 2nd typical variable of the set Y: Y2 = −4.538 × Thickness+1.995 × Porosity–1.112 × Compression rebound rate.
Equation for the 1st typical variable of the set X: X1 = −0.23 × filtration resistance −0.003 × air permeability −0.035 × filtration efficiency.
Formula for the 2nd typical variable of the set X: X2 = −0.126 × filtration resistance +0.01 × air permeability +0.141 × filtration efficiency.

These equations are derived from Supplementary Tables 8, 9.

Take the analysis of the first typical variable as an example: From Supplementary Table 7, we know that the correlation coefficient of the first pair of typical variables reaches 0.946, *P* << 0.05, which means that there is a significant strong positive correlation between the first typical variables. From the first typical variable relationship equation above, we know that if the Thickness of Y1 increases, then Y1 increases, then X1 also increases, and X1 increases, then the product performance will decrease.

Similarly, there are similar increasing and decreasing relationships among other typical variables.

**FIGURE 5:**
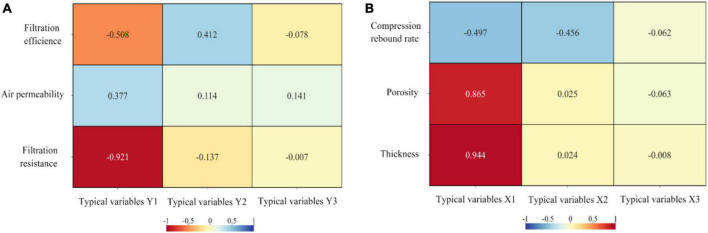
Thermal diagram of typical variable. **(A)** Y cross-load matrix. **(B)** X cross-load matrix.

From the Supplementary Table 10, it can be seen that the typical variable X1 explains 70.226% of the information content of the indicator in set Y and 46.437% of the information content of the indicator in set X. The typical variable X2 explains 19.021% of the information content of the indicator in set Y and 18.337% of the information content of the indicator in set X. The typical variable X2 explains 19.021% of the information content of the indicator in set Y and 18.337% of the information content of the indicator in set X. The typical variable Y1 explains 41.583% of the information content of the indicator in set Y and 62.886% of the information content of the indicator in set X. The typical variable Y2 explains 41.583% of the information content of the indicator in set Y and 62.886% of the information content of the indicator in set X. The typical variable Y2 explains 6.707% of the information content of the indicator in set Y and 6.957% of the information content of the indicator in set X. The heat diagram of typical variable Y/X cross load matrix can be seen in Figure 5.

## 4. Multiple regression analysis and establishment of predictive models

### 4.1. Establishing linear regression models between process parameters and structural variables

#### 4.1.1. Linear regression general equation

The model for the multiple linear regression analysis (Qin, 2020) is:


(2) $$\left\{ \begin{array}{c} y=\beta_{0}+\beta_{1}\,x_{1}+\beta_{2}\,x_{2}+\ldots +\beta_{m}\,x_{m}+\varepsilon \\ \varepsilon ∼N\,\left(0,\delta^{2}\right), \end{array}\right.\;m=1,2,\ldots \,n$$

where β_0_, β_1_, β_2,_…, β_*m*_, δ^2^ is the partial regression coefficient, which is not correlated with *x*_1_, *x*_2_…*x*_*m*_ is no correlation, and ε is the random error term. Suppose, there is a linear relationship between the dependent variable and the respective variable, then the linear overall regression model between them can be expressed as: *y* = β_0_ + β_1_*x*_1_ + β_2_*x*_2_ + ε, where ε is the random error term, ε∼N(0,δ)2.

#### 4.1.2. Multiple linear regression solution results

The multiple regression equations between Thickness (y_1_), Porosity (y_2_), Compression rebound rate (y_3_) and receiving distance (x_1_), hot air velocity (x_2_) were obtained using Matlab’s regression function.


(3) $$\left\{ \begin{array}{c} y_{1}=-0.932+0.0396\,x_{1}+0.0013\,x_{2}\\ y_{2}=80.004+0.2072\,x_{1}+0.0061\,x_{2}\\ y_{1}=77.4328+0.1487\,x_{1}-0.0009\,x_{2} \end{array}\right.$$

**FIGURE 6:**
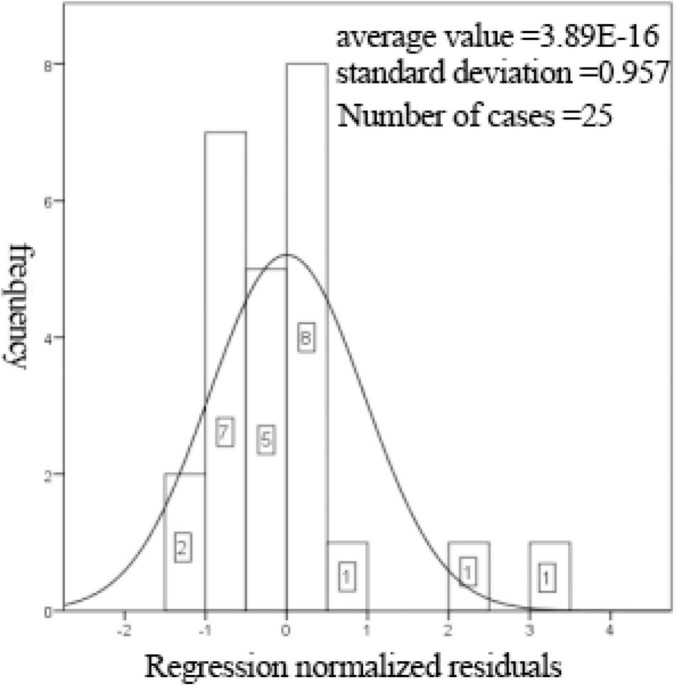
Histogram of regression-standardized residuals with normal curve.

**FIGURE 7:**
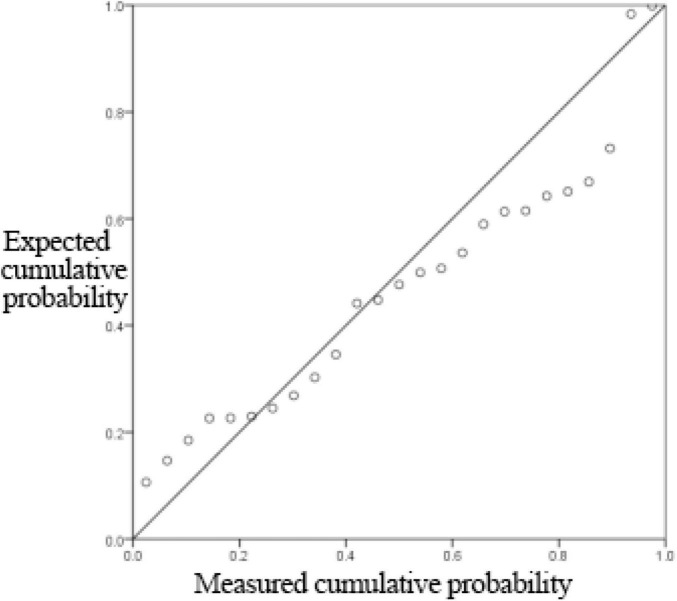
Normal P-P plot of regression-standardized residuals.

Supplementary Table 11 shows that the linear regression fitting effect of thickness to receiving distance and hot air velocity is better (*R*^2^ = 0.784), indicating that the receiving distance and hot air velocity can explain 78.4% of the change of thickness index, which is generally explained. Figures 6, 7 show that the residuals roughly follow the normal distribution, indicating that the model is generally established.

Here is an example of analyzing the multiple linear regression equation for Thickness.

### 4.2. Establishing non-linear regression models between process parameters and structural variables

#### 4.2.1. Non-linear regression general equation

The multiple non-linear regression (Luyu and Jing, 2021) equation model is:


(4) $$\left\{ \begin{array}{c} y=\beta_{0}+\beta_{1}\,x_{i}+\beta_{2}\,x_{i}^{2}+\varepsilon \\ \varepsilon ∼N\,\left(0,\delta^{2}\right), \end{array}\right.\;i=0,1,2,\ldots \,n$$

There is a non-linear relationship between the dependent variable and the respective variable, then the non-linear overall regression model between them can be expressed as : $y=\beta_{0}+\beta_{1}\,x_{i}+\beta_{2}\,x_{i}^{2}+\varepsilon$, where ε is the random error term, ε∼N(0,δ)2.

#### 4.2.2. Multiple non-linear regression solution results

The above non-linear regression model is solved using Matlab’s nlinfit function.

**FIGURE 8:**
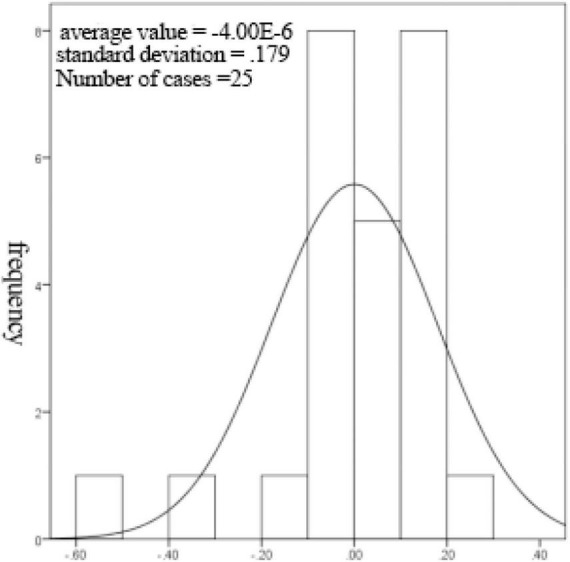
Histogram of regression normalized residuals with normal curve.

**FIGURE 9:**
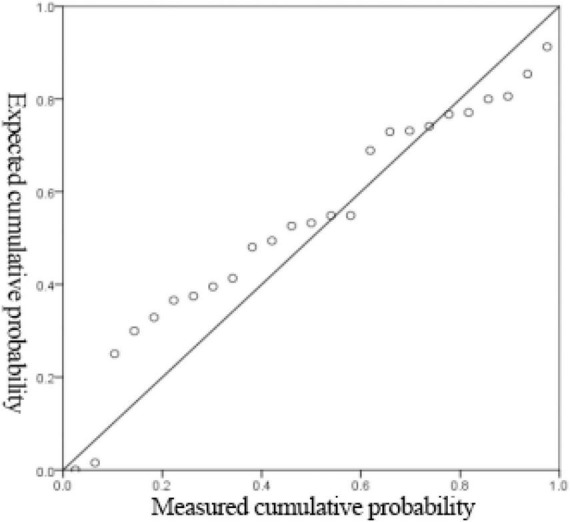
Normal P-P plot of regression normalized residuals.

From Supplementary Table 12, it can be seen that the non-linear regression fitting effect of thickness to receiving distance and hot air velocity is better (*R*^2^ = 0.782), indicating that the receiving distance and hot air velocity can explain 78.2% of the change of thickness index, and the degree of interpretation is average. Figures 8, 9 show that the residuals roughly follow the normal distribution, indicating that the model is generally established.


(5) $$\left\{ \begin{array}{c} y_{1}=-0.30836+0.03956\,x_{1}+6.27802690637809*10^{-7}\,x_{2}^{2}\\ y_{1}=83.06234+0.20724\,x_{1}+3.00946686674695*10^{-6}\,x_{2}^{2}\\ y_{1}=77.26178+0.14872\,x_{1}-6.71549591749362*10^{-7}\,x_{2}^{2} \end{array}\right.$$

Here the multivariate non-linear regression equation for the analysis of Thickness is used as an example:

In summary, there is no obvious difference between the two models according to the goodness-of-fit comparison of the two models, and the multiple linear regression model is actually better than the non-linear regression model in terms of mean squared error, so for the quantitative analysis of thickness, porosity, compression resilience and process parameters, multiple linear regression can be satisfied, and can also be used for prediction. However, due to the pursuit of higher model accuracy, the BP neural network model is introduced next.

### 4.3. Back propagation neural network prediction model for structural variables

#### 4.3.1. Selection of network structure

The receiving distance and hot air velocity in the process parameters are used as independent variable inputs. Thickness, Porosity and Compression rebound rate are used as dependent variable targets, respectively. The uninterpolated sample data in data 1 are divided into three categories of training set (70%), test set (15%), and test set (15%).

(1) The number of hidden neurons applicable to each dependent variable was derived from many experiments. Finally, the number of hidden neurons for Thickness and Porosity was determined to be four, and the number of hidden neurons for Compression rebound rate was three.

(2) The training method for the neural network model is the Levenberg–Marquardt algorithm.

#### 4.3.2. Test for each parameter (to analyze thickness as an example)

(1) R coefficients for training set, test set, validation set and all data.

**FIGURE 10:**
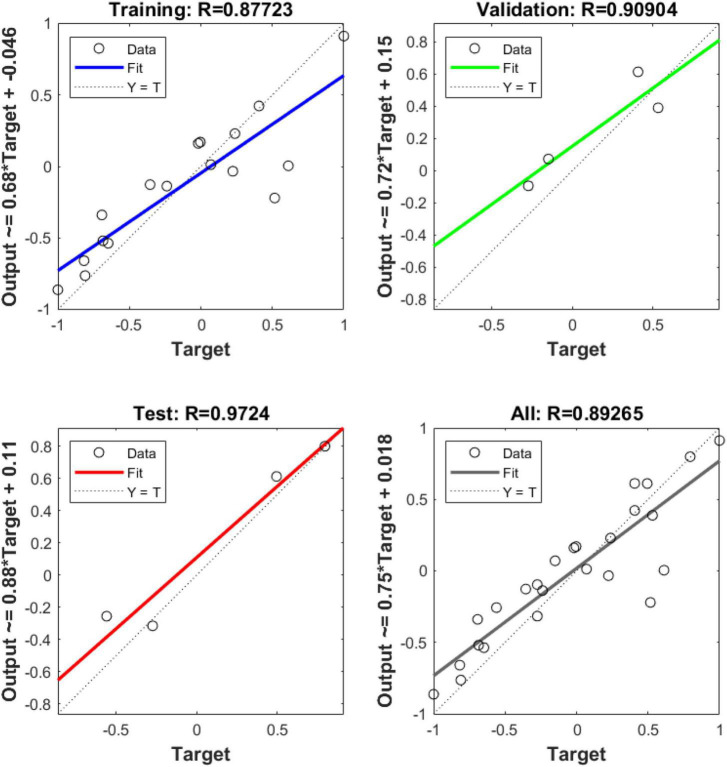
R-factor.

The regression value R indicates the correlation between the predicted output and the target output, and it can be seen from the figure below that the *R* values are all greater than 0.85, indicating a good fit. It can be seen from Figure 10.

(2) Mean Squared Error

**FIGURE 11:**
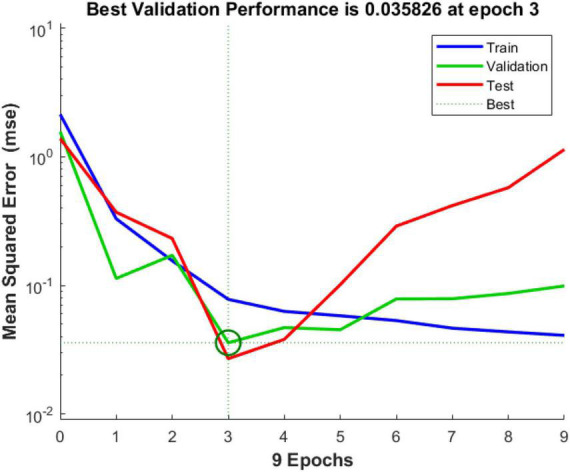
Optimal training effect of the 3rd generation.

The green circle in the figure shows the number of iterations and the mean square error size of the network at the best mean square error of the validation set, which is 0.0358, and the final iteration number of 3. It can be seen from Figure 11.

(1) Historical residuals of training data

**FIGURE 12:**
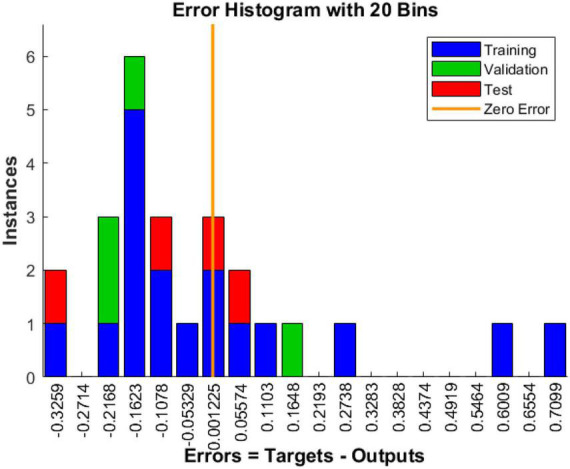
Error histogram.

The error histogram can be spent on the error between the predicted output and the target output. It can be seen from Figure 12.

With the BP neural network result plots of (1–3), we can consider this model training result acceptable.

According to Supplementary Tables 11, 12 and Figures 10–12, the goodness-of-fit of multiple linear regression and multiple non-linear regression is not as high as that of BP neural network model, and the mean squared error of multiple linear regression and multiple non-linear regression is higher than that of BP neural network model. Therefore, using this prediction model, the prediction of structural variables for a given process condition has positive practical significance.

## 5. Establishment of BP neural network prediction model with maximum filtering efficiency

### 5.1. Establishment of BP neural network

(1) Similar to the BP neural network model above, the process parameters, structural variables and product performance in filtration resistance and filtration efficiency are used here as independent variables input and filtration efficiency as dependent variable target, and the 75 sample data in data three are divided into Three categories, training set (70%), test set (15%), and test set (15%) (Kessler et al., 2007; Liu et al., 2014).

(2) The number of hidden neurons applicable to the dependent variable is derived through many experiments, and the best-hidden neuron is finally determined to be five.

(3) The training method of neural network model is Levenberg-Marquardt algorithm.

### 5.2. Back propagation neural network model parameter test

**FIGURE 13:**
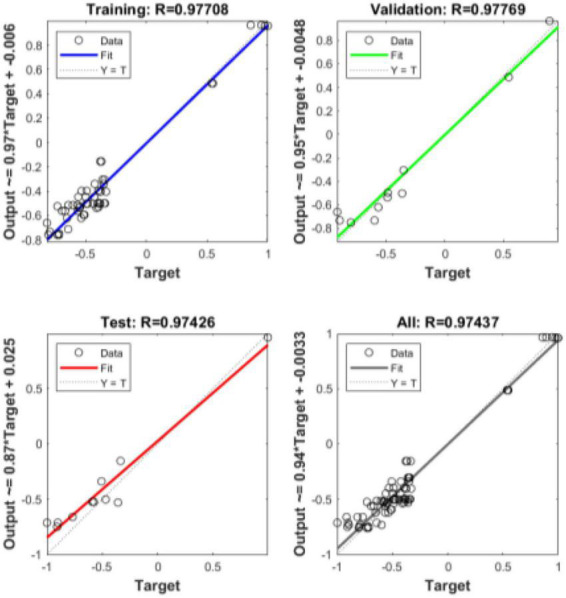
R-coefficient.

**FIGURE 14:**
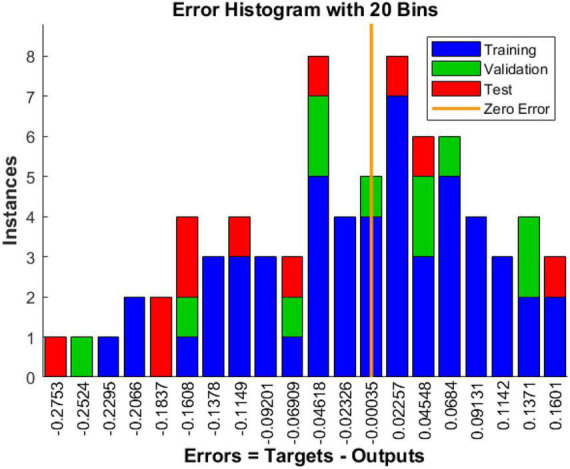
Historical residuals of training data.

From the Figures 13, 14, it can be seen that the results of this model training are acceptable.

### 5.3. Back propagation neural network to find the maximum filtration efficiency

Using the obtained training model, different value pairs of hot air velocity and receiving distance of the process parameters are carried out to iteratively solve the problem to find the maximum filtration efficiency. Here we expand the range of receiving distance values to 20–60 cm, and the value of hot air velocity to 800–2,500 r/min, pair different process parameters, and predict the maximum filtration efficiency. The results of the iterations are shown in the Supplementary Table 13.

The result shows that the maximum filtration efficiency is 81.904%, which corresponds to the process parameters of receiving distance 20 cm, hot air velocity 1,250 r/min or 1,280 r/min.

## 6. Conclusion

There are many technological parameters in the preparation of intercalated melt blown non-wovens, among which there are cross effects. These parameters are more complex when the intercalated air flow is added. This article extracts the attribute values of process variables, structural parameters, product performance, and other attributes from the open dataset. It analyzes the relationship between variables through descriptive statistics and multiple regression. It is found that the BP neural network model is more accurate in predicting structural variables. Therefore, using the BP neural network prediction model has good guiding significance for improving the product performance of intercalated meltblown non-woven materials. In addition to this problem, the model can also adapt to too many other multi factor analysis problems.

## Data availability statement

The original contributions presented in this study are included in this article/Supplementary material, further inquiries can be directed to the corresponding author.

## Author contributions

HX was responsible for putting forward the ideas and responsible for the structure, writing and revision of the entire manuscript, and provided the funds. J-WX, L-XY, and Y-TY were mainly responsible for the programming and mathematical derivation. Z-QC was mainly responsible for the language revision and technical discussion of the manuscript. All authors contributed to the article and approved the submitted version.

## Supplementary material

Supplementary Table 1 Before intercalation.

Supplementary Table 2 After interpolation.

Supplementary Table 3 Analysis results of correlation analysis. ***, **, and * represent 1, 5, and 10% of Sig. value, respectively.

Supplementary Table 4 Normality test. ***, **, and * represent 1, 5, and 10% of Sig. value, respectively.

Supplementary table 5 Correlation analysis of structural variables. ***, **, * represent 1%, 5%, 10% of Sig. value respectively.

Supplementary Table 6 Product performance related analysis. ***, **, and * represent 1, 5, and 10 of Sig. value, respectively.

Supplementary Table 7 Typical correlation coefficients. *** and ** represent 1 and 5% of Sig. value, respectively.

Supplementary Table 8 Typical coefficients of variables for set Y.

Supplementary Table 9 Typical variable coefficients for set X.

Supplementary Table 10 Variance interpretation ratio.

Supplementary Table 11 Analysis of fitting results.

Supplementary Table 12 Analysis of the fitting results.

Supplementary Table 13 Partial filtration efficiency iteration results. The result shows that the maximum filtration efficiency is 81.904%, which corresponds to the process parameters of receiving distance 20 cm, hot air velocity 1,250 r/min or 1,280 r/min.
